# Resolving and Analyzing Landfast Ice Deformation by InSAR Technology Combined with Sentinel-1A Ascending and Descending Orbits Data

**DOI:** 10.3390/s20226561

**Published:** 2020-11-17

**Authors:** Zhiyong Wang, Jian Liu, Jinning Wang, Lihua Wang, Meng Luo, Zihao Wang, Ping Ni, Hao Li

**Affiliations:** College of Geodesy and Geomatics, Shandong University of Science and Technology, Qingdao 266590, China; CYlovepeach@139.com (J.L.); wjn2018sk@163.com (J.W.); 17854253873@139.com (L.W.); mengluowest@163.com (M.L.); wangzh9697@163.com (Z.W.); niping1217@163.com (P.N.); lh1769176730@gmail.com (H.L.)

**Keywords:** InSAR, deformation measurement, fast ice, Baltic Sea, Sentinel-1A

## Abstract

Detailed mapping of landfast ice deformation can be used to characterize the rheological behavior of landfast ice effectively and to improve sea ice modeling subsequently. In order to analyze the characteristics, trends and causes of deformation comprehensively and accurately, the Sentinel-1A ascending and descending orbits data were used to detect the horizontal and vertical deformation of the fast ice in the Baltic Sea. Firstly, the fast ice edge lines were acquired through feature extraction with interferometric coherence images and SAR amplitude images. Then, the deformation transformed model was constructed according to the geometric relationship of multi-orbits deformation measurements. Finally, the landfast ice deformations were resolved and the horizontal and vertical deformations were obtained. The results showed that the maximum deformation was—44 cm in horizontal direction and 16 cm in vertical direction within the fast ice region of 960 km^2^ during the time from 2 to 16 February 2018. The southwest wind was the principal reason for the deformation, which made the deformation mainly occur in the horizontal direction from east to west. Moreover, the inner fast ice kept stable due to the protection of outer consolidated ice. The results showed that the deformation trend and characteristics can be better understood by using InSAR technology that was combined with multi-orbits SAR data to resolve and analyze the landfast ice deformation.

## 1. Introduction

The change of sea ice, a sensitive indicator to global climate change, is closely related to the regional ecological environment, production and development in polar and high latitude regions. Landfast ice is a kind of stable sea ice that grows along the coast, islands, reefs and grounding ice ridges and is firmly attached to them. The growth, development and deformation of landfast ice not only affect the thermal balance of the marine environment but also affect the temperature and salinity cycle of the ocean. The stability and area of landfast ice are affected by the depth, temperature and salinity of sea water or other comprehensive factors [1,2]. During the ice season of high latitudes (e.g., the Arctic and the Baltic Sea), multi-national resource explorers will create ice roads on sufficient thickness landfast ice for the transport of large instruments and resources [3,4]. In mid-latitude regions (such as Liaodong Bay of China), the thickness, area, stability and lasting time of fast ice are smaller than those in high-latitude regions and its existence mainly affects the safety of some offshore structures (e.g., ships and ports). Generally, fast ice is only subjected by the rise and fall of sea level, which will result in a small vertical movement rather than large horizontal displacements. However, the surface and interior of landfast ice may deform from a few centimeters to tens of centimeters under the driving of external factors such as ocean current, strong wind and ice floes impact. Ice cracks will occur when the stresses exceed the yield stresses, which greatly threaten the personal safety. It is a powerful basis that monitoring the deformation of fast ice for evaluating and predicting the stability of sea ice. On the one hands, the drift direction of sea ice can be obtained according to the deformation trend of fast ice, which provides a reliable guidance for the route planning and risk aversion of marine transportation. On the other hand, the accurate inversion of fast ice displacement value and deformation stress can effectively evaluate the stability of ice road, and provide early warning and guarantee for the safety of transportation on sea ice. Therefore, it is very significant to detect the deformation, evaluate the possible destructive events of fast ice, and provide effective warning for local production and life in time and accurately.

It has always been a difficult issue that how to accurately obtain the deformation of fast ice over large area while ensuring the immediate effectiveness and economy of the method [5]. Most of fast ices exist in polar or high-latitude sea, which is difficult to measure the sea ice parameters in the field due to the unique and complex terrain. The remote sensing technology can effectively achieve the sea ice parameters. However, due to the influence of weather such as clouds and fog, optical remote sensing cannot effectively obtain the measurement in time, and the resolution of radiometer is low. Radar altimeter can inverse the thickness of sea ice with centimeter level accuracy [6], but the traditional radar altimeter has a poor spatial resolution and narrow swath due to the limitation of nadir looking, so it still includes many limitations in sea ice study. As SAR (Synthetic Aperture Radar) can obtain large-area and high-resolution images with all-weather imaging capability and day/night data acquisition, it is more suitable to detect the sea ice.

InSAR (Synthetic Aperture Radar Interferometry) technology has been proved to be able to obtain centimeter-scale deformation of landfast ice [3,4,7,8]. Dammert, P.B.G. et al. [9] have demonstrated that InSAR technology has an important role and application prospect in studying the backscattering characteristics of stationary fast ice and the small deformation of scattering sources in Bothnia Bay of the Baltic Sea. The survey found that the maximum displacement occurred on the ice sheet cut off by the tracks of two icebreakers, and the deformation value was 94 cm. InSAR technology can discriminate the fast ice accurately. Meyer, F.J. et al. [10] have obtained the boundary line and variation characteristics of landfast ice in the Seward Peninsula, Alaska for 46 days by the InSAR results of ALOS PALSAR data, which proved that InSAR technology with L-band data can map landfast ice with high accuracy and robustness in various environmental conditions. In terms of deformation factors and fringe features, Berg, A. et al. [7] have found that precipitation and temperature were likely to be responsible for the decrease of coherence in fast ice and the impacting of drift ice would cause a great impact on the boundary of fast ice to produce dense fringes. Marbouti, M. et al. [8] have successfully got the deformation trend and cause of fast ice by Sentinel-1A data in Baltic Sea. In order to estimate the deformation trend of fast ice more accurately, Dammann, D.O. et al. [3,4] have proposed an inverse model based on matching the direction of interferometric fringes, which divided the deformations into 5 modes and successfully evaluated the potential dangers of the ice roads and the possibility of ice surface cracks. However, due to the influence of the resolution of inverse model and time baseline, the detection of deformations may be estimated incorrectly.

Based on the above, there are two main problems in the detection of fast ice deformation. (i) How to obtain the region of fast ice accurately? The fast ice was separated from drift ice by long time baseline in [10]. Although the boundary of fast ice was extracted effectively, the deformation characteristics of fast ice could not be accurately reflected due to the long time span. The region was acquired by the coherence images in [8], but the coherence is easily affected by temperature, precipitation, snow and other weather factors. (ii) How to estimate the deformation characteristics of fast ice accurately? The surface deformations are three-dimensional, while InSAR deformation measurements are one-dimensional (Line-Of-Sight direction, LOS). Deformations were divided from one-dimensional results into five different modes in [3,4], while ignoring the condition that horizontal and vertical deformation existed simultaneously. At present, most satellites operate in near-polar solar orbit and the deformation of azimuth direction (perpendicular to LOS) will be ignored [11]. In addition, the InSAR LOS measurements are most sensitive to ground uplift or subsidence because of the side-looking geometry of SAR sensor and its generally low incidence angle. Usually, the estimation of vertical deformation was based on the assumption that there was no horizontal displacement [12,13,14], however, which would likely to wrongly deal with the condition that both of them existing. In recent years, InSAR technology has been widely used in the reconstruction of seismic 3D deformation field and 3D surface displacement measurement by using ascending and descending orbits data and combining with MAI (Multi-aperture InSAR) technique, GPS measurement or Offset-stacking technology [15,16,17,18]. However, the researches on resolving the deformations of fast ice using InSAR are relatively few. Therefore, in order to avoid the wrong estimation of deformation types and obtain the right displacement results, this study utilized the ascending and descending orbits data to detect the horizontal and vertical deformations of fast ice.

In this paper, the deformations of fast ice were acquired by InSAR technology with combining the ascending and descending orbits data of Sentinel-1A. The boundary line of fast ice was automatically obtained by using the texture features of coherence and amplitude images, and then the deformation in LOS was transformed into the horizontal and vertical displacement according to the geometric relationship of multi-orbits deformation measurements. On this basis, the deformation trend and reasons of fast ice were estimated accurately. In Section 2, the study area and data were introduced in detail. In Section 3, the processing flow of interferometric SAR data, the algorithm to extract fast ice boundary and the deformation transformed model were described. In Section 4, the results were showed, including coherence images, interferograms and deformation maps. In Section 5, discusses and analyses were carried out. The conclusions of this study, the developments and potentials of InSAR technology in studying sea ice in feature were outlined in Section 6.

## 2. Data and Materials

**Figure 1:**
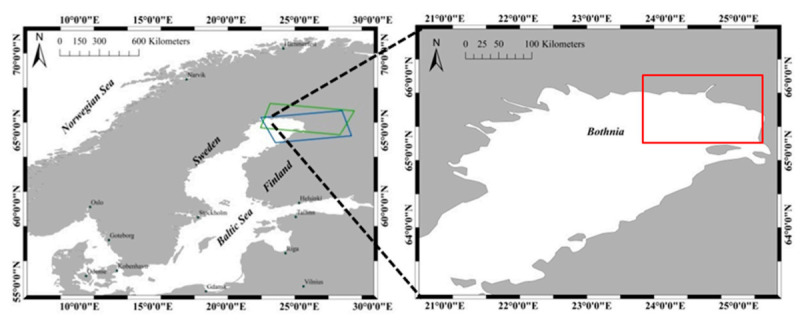
Study area: the blue and green boxes represent the coverage of the images of ascending and descending orbits respectively; and the red rectangle represents the study region.

The study area is located in the north part of the Bothnia Bay of Baltic Sea (22.5°–25.5° E, 65.0°–65.8° N), as shown in Figure 1. The Baltic Sea is a semi-enclosed sea in the north of Europe. Fast ice grows along the coastline in Bothnia Bay in November firstly, and it will rapidly expand by attaching coastlines, islands, reefs and grounding ice ridges. The growth of fast ice is limited by water depth, and the water depth range of fast ice growth generally does not exceed 5–15 m due to the influence of dynamic forces [19]. The ice season of Baltic Sea generally lasts 5–7 months, from November of the previous year to May of the next year. The fast ice of Bothnia Bay usually lasts throughout the ice season, as the salinity is lower (less than 0.5‰ [7]) than other sea regions. In order to avoid a series of disasters caused by sea ice, the monitoring of fast ice has always been the focus of attention in this area.

Four SLC (Single Look Complex) images in IW mode acquired from Sentinel-1A were selected. The specific information of Sentinel-1A data were shown in Table 1. The study images were acquired in February 2018 and their characteristics were shown in Table 2. Since the co-polarization data of C-band was more sensitive to the dielectric constant and surface roughness of ground objects than cross-polarization [20] and considering the influence of snow cover, VV polarization data were better than HH polarization to study sea ice in this paper [21].

The interferometric parameters were shown in Table 2. Both of image pair A and B has the same time baseline of 12 days, and the time difference between the two master images was 2 days. The same time baseline and similar imaging time ensured the consistency of experimental results.

Moreover, the ACE2 (Altimeter Corrected Elevations, v2) DEM released by NASA (National Aeronautics and Space Administration) was applied to InSAR data processing in this study [23]. In this paper, the daily ice chart published by the Swedish Meteorological and Hydrological Institute (SMHI) was used as reference data for classification of sea ice types [24]. As auxiliary data, a lot of meteorological information was collected such as precipitation, temperature, winds and sea level during the imaging period. These data were mainly released by the meteorological observation station of Kemi Ajos (24.52° E, 65.67° N) [25,26].

## 3. Methods

**Figure 2:**
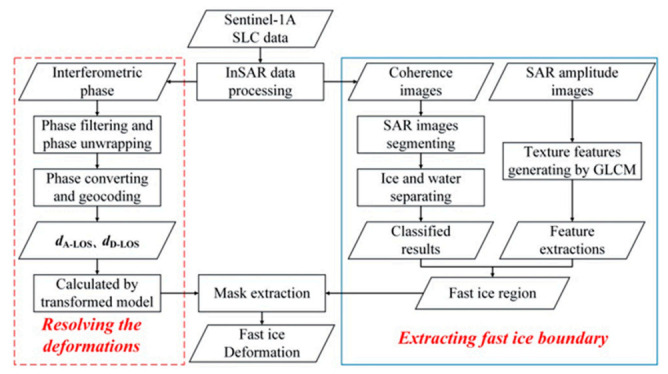
Methodology Workflow: it mainly consists of two parts, extracting fast ice boundary and resolving the deformations.

As shown in Figure 2, combining with the ascending and descending orbits data, a method was established to analyze the deformation of landfast ice in this paper. Firstly, the coherence images and fringe patterns were obtained through interferometric processing, and the sea ices were separated from sea water by using coherence images. In order to accurately determine the region of fast ice, several texture features were calculated by SAR amplitude images through the GLCM (gray level co-occurrence matrix). Then, the edge line of fast ice was obtained by object-oriented feature extraction. Secondly, the deformation transformed model was established according to the geometric relationship of multi-orbits deformation measurements, and the deformations of LOS direction were transformed into horizontal and vertical displacements. It should be noted that the north-south deformation of the fast ice was ignored in the paper. Finally, the deformation results of fast ice were obtained by using mask data. According to these results, the deformation trend and reasons were analyzed deeply.

### 3.1. InSAR Data Processing

The fundamentals of InSAR technology can be found in reference [27,28]. First, the baseline estimation was counted by using the master and slave images of the same orbit. Then, the master and slave images were co-registered at sub-pixel level using Sentinel-1A precise orbit data and external DEM data, and speckle noises were suppressed by multi-looking processing with the azimuth and range looks were 1 and 5 respectively. To further remove speckle noises, the Lee [29] algorithm with filtered window of 5 × 5 was used to filter SAR images. Next, the master and slave images after co-registered were conjugate multiplied to generate interferograms and coherence images. In order to optimize the phase unwrapping results and improve the visibility of the fringes, the flat phase was removed with the rough DEM and the Goldstein algorithm [30] was adopted for phase filtering in this study. After phase filtering, the minimum cost flow (MCF) algorithm [31] was used into phase unwrapping. Finally, the unwrapped phase was converted into displacement and geocoded, and the SAR image coordinate system was transferred to the mapping coordinate system (WGS_1984_UTM_Zone_34N).

### 3.2. Extracting Fast Ice Boundary

The complex coherence γ between two complex SAR images S1 and S2 is defined as [10]:

(1) $$\gamma =\frac{\left\langle s_{1}s_{2}^{*}\right\rangle }{\sqrt{\left\langle s_{1}s_{1}^{*}\rangle \times \langle s_{2}s_{2}^{*}\right\rangle }}\;0\leq \left|\gamma \right|\leq 1$$

where <…> denotes spatial averaging operation. The coherence value represents the stabilities and correlations of ground objects during the acquisition period of master and slave images. For fast ice, the coherence value could be high or low, which mainly depends on the actual situation of the ground surface and the parameters of sensors. Generally, the coherence value γ consists of three parts [7], i.e.,

(2) $$\gamma =\gamma_{Thermal}\times \gamma_{Spatial}\times \gamma_{Temporal}$$

where *γ_Thermal_* is the decorrelation caused by thermal noise of the receiver, which can be determined by the signal-to-noise ratio (SNR). *γ_Spatial_* is the decorrelation caused by the difference of incidence angles and proportional to the vertical baseline, so it is also known as baseline decorrelation [10]. *γ_Temporal_* is the correlation factor associated with incoherent changes in the scattering medium in interval period. Compared with the two decorrelation factors previously mentioned, *γ_Temporal_* characterizes with large variation and poor stability, which is the major factor of decorrelation and the principal quantity measured. In the study of fast ice, the numerical variation of *γ_Temporal_* is caused by the movement of ice floes or the change of surface scatterer within a resolution cell [7].

The fast ice boundary was obtained by combining coherence image and SAR amplitude. The kernel of the algorithm is to get the rough fast ice boundary by coherence image, and then get the accurate boundary through the texture features obtained from the amplitude image. Firstly, SAR images should be segmented and the data processing would be based on the segmentation units [32]. In this paper, the multi-resolution segmentation algorithm was used to segment the coherence images. Secondly, the threshold value was determined by Otsu algorithm [33] to obtain the sea ice region with high coherence value. Finally, several texture features were achieved by SAR amplitude images through GLCM operation, and the fast ice edge line was obtained by object-oriented feature extraction. A coastline data released by the GSSHG (Global Self-Consistent Hierarchical, High-resolution Geography database) of the NOAA (National Oceanic and Atmospheric Administration) was utilized as a land mask or separating the sea and the land, and it also serves as the boundary line of the fast ice near the land side [34].

### 3.3. Calculating the Horizontal and Vertical Deformation

**Figure 3:**
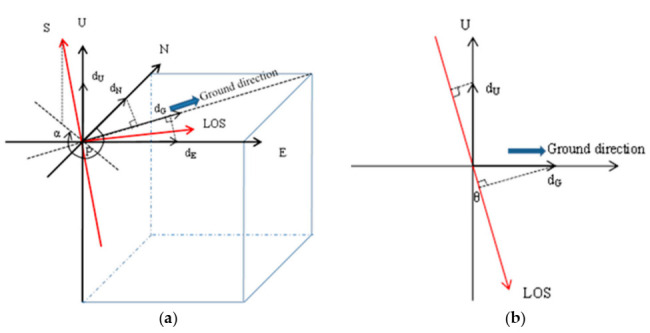
(**a**,**b**) The three-dimensional and two-dimensional schematic diagram of ground surface deformation under Sentinel-1A ascending orbits mode, respectively.

Figure 3 is a three-dimensional schematic diagram of ground surface deformation under Sentinel-1A ascending orbits mode. Coordinate axes S, U, N, E and LOS represent the azimuth, vertical, north-south, east-west and radar line of sight direction respectively. The deformation variables in vertical, north-south, east-west and ground direction are represented by *d_U_, d_N_, d_E_*, and *d_G_* respectively. *α* is the azimuth angle of satellite orbit (positive clockwise from north), and *θ* is the incidence angle of radar pulse signal. Firstly, according to the geometric relationship, the deformation in the north-south and east-west directions were decomposed into the ground distance direction, i.e.,

(3) $$d_{G}=d_{N}\cos\left(\alpha_{A}-\frac{3\pi }{2}\right)+d_{E}\sin\left(\alpha_{A}-\frac{3\pi }{2}\right).$$

Then, the deformations in the vertical and ground directions were decomposed into LOS direction by Equation (4)

(4) $$d_{A\_LOS}=d_{U}\cos\theta_{A}-d_{E}\sin\theta_{A}\sin\left(\alpha_{A}-\frac{3\pi }{2}\right)-d_{N}\sin\theta_{A}\cos\left(\alpha_{A}-\frac{3\pi }{2}\right)$$

where *d_A_LOS_, α_A_* and *θ_A_* represent the deformation in LOS direction, the azimuth angle of ascending orbit and the incidence angle of radar under ascending mode respectively. In the same way, under the descending orbit mode, the deformation in LOS direction, *d_D_LOS_*, can be obtained by Equation (5)

(5) $$d_{D\_LOS}=d_{U}\cos\theta_{D}-d_{E}\sin\theta_{D}\sin\left(\alpha_{D}-\frac{3\pi }{2}\right)-d_{N}\sin\theta_{D}\cos\left(\alpha_{D}-\frac{3\pi }{2}\right).$$

Since the near-polar orbit radar satellite is not sensitive to the displacement of the north-south direction, it is difficult to obtain the deformation in this direction [35,36,37]. Due to Sentinel-1A with a near-polar sun synchronous orbit, the deformations in the north-south direction of the fast ice were regarded as 0. The deformation in the east-west and vertical directions could be resolved by combining Equations (4) and (5) [11,15,16], i.e.,

(6) $$\left[\begin{array}{c} d_{U}\\ d_{E} \end{array}\right]={\left[\begin{array}{cc} \cos\theta_{A} & -\sin\theta_{A}\sin\left(\alpha_{A}-\frac{3\pi }{2}\right)\\ \cos\theta_{D} & -\sin\theta_{D}\sin\left(\alpha_{D}-\frac{3\pi }{2}\right) \end{array}\right]}^{-1}\left[\begin{array}{c} d_{A\_LOS}\\ d_{D-LOS} \end{array}\right].$$

## 4. Results

The main research results included coherence images, interferograms and deformation maps. The coherence value reflected the stability and correlation of sea ice during the interval time when the interferometric pair images were acquired. The interferograms reflected the features of fast ice deformations. The denser the fringes are, the more serious the deformations are. The deformation maps directly illustrate the displacement of fast ice.

### 4.1. Coherence Images

**Figure 4:**
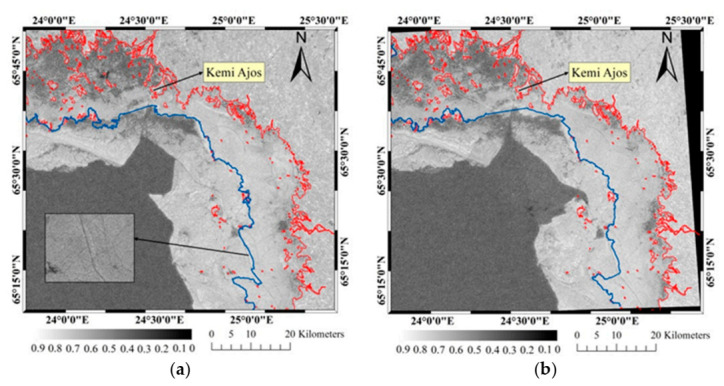
(**a**,**b**) The coherence images obtained by image pair A and image pair B respectively. The blue and red curves were the fast ice edge lines obtained by the method proposed in this paper and the coastline or islands released by NOAA, respectively.

The coherence images were shown in Figure 4, where the red curve is the coastline and islands obtained by NOAA data [34]. Figure 4a was the coherence image obtained by image pair A, and the whole mean coherence value was about 0.43. In the ocean region, there was an obvious difference of coherence values between the sea ice (bright area) and sea water (dark area). The coherence values determined by image pair B were shown in Figure 4b and the average value was about 0.39.

### 4.2. Interferograms

**Figure 5:**
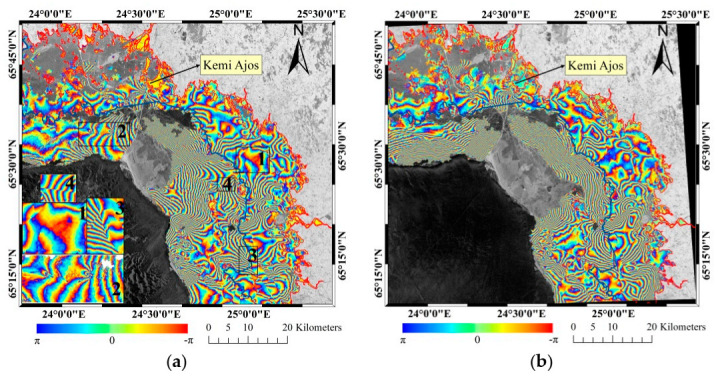
(**a**,**b**) The filtered interferograms obtained by image pair A and B respectively. The SAR amplitude images were used as background images. The blue and red curves represented fast ice boundary and coastline, respectively.

The filtered interferograms were displayed in Figure 5, and the change of each color cycle represents that the displacement change in LOS is about half wavelength length. Compared with Figure 5a, the density of fringe increased in some regions of Figure 5b, but the variation trend of the whole fringe density was consistent. That is to say, the closer to the coast the sparser interferometric fringes were. It also illustrated that the peripheral sea ice acts as a protective barrier for the interior fast ice.

### 4.3. Deformation Maps

**Figure 6:**
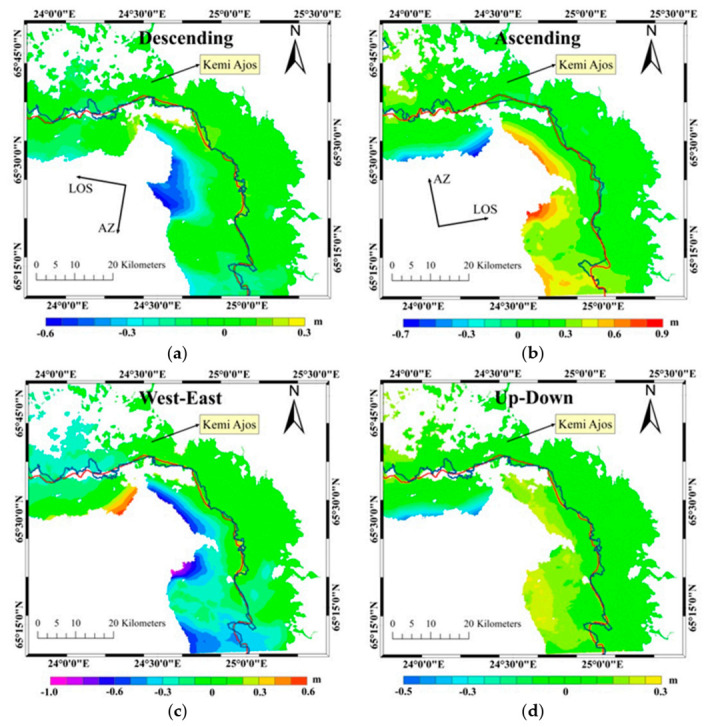
(**a**,**b**) The deformation in LOS obtained by image pair A and B respectively. (**c**,**d**) The horizontal and vertical deformation obtained by the deformation transformed model respectively. The blue and red curves were the fast ice edge lines obtained by the method proposed in this paper and the SMHI ice chart, respectively.

The displacements in LOS direction determined by the descending orbits data was displayed in Figure 6a, and the negative or positive sign represents the movement away from (sink or move to west) or close (uplift or move to east) to the satellite. Figure 6b showed the displacements in LOS direction got by image pair B, but the same signs stood for opposite deformation directions to that of Figure 6a. The horizontal and vertical deformation calculated by Equation (6) were shown in Figure 6c,d, and the positive and negative signs represented the displacements in east and west or subsidence and uplift, respectively.

## 5. Discussions and Analysis

### 5.1. Factors of Decorrelation

**Figure 7:**
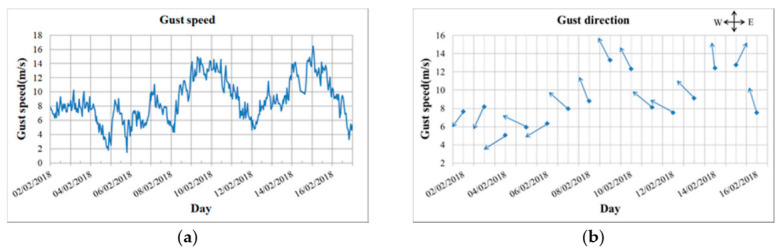
(**a**) Gust speed in hour; (**b**) Gust direction and average speed in daily.

In the coherence images, the value decreased in the area near shore significantly, due to the drastic change of scattering geometry and the shallow water that made fast ice grounded and ice ridges formed [7]. The values in the upper right corner of coherence images were also low because this area was the estuary of rivers, in which the sea water was high fluidity and the warm river water raised the temperature of the sea water. There were some obvious line features with low coherence value in the inner part of fast ice, which were mainly caused by artificial ice roads, as shown in Figure 4a. It had some great differences at the boundary of sea ice between two coherence images due to the influence of winds. As shown in Figure 7b, the wind direction changed from southeast to southwest and winds changed the drift direction of ice floes, which caused serious impact and compression on fast ice. Correspondingly, the interferometric fringes also changed greatly in the region where the coherence value changed.

**Figure 8:**
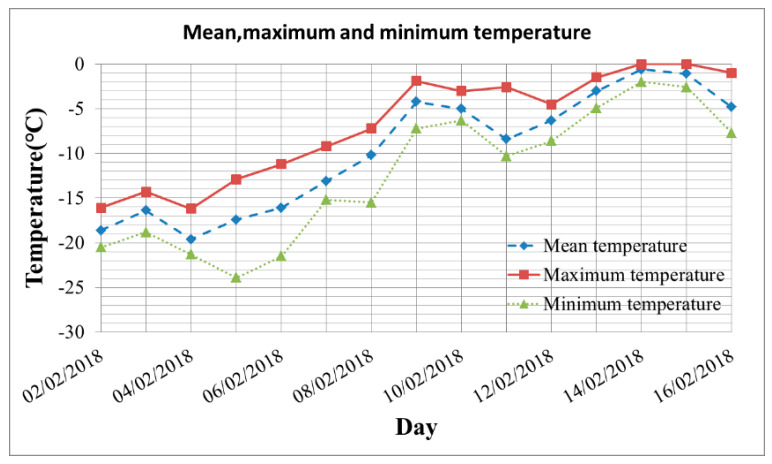
Temperature information: the minimum and maximum temperatures were −23.9 °C and 0 °C respectively, and the overall trend was upward.

The factors for decorrelation can be roughly attributed to temperature, precipitation and wind. First, the temperature difference between the acquisitions of tow images within image pair A was so large, as shown in Figure 8, the temperature raised from −19 °C to −0.9 °C. When the temperature was lower than −5 °C, the low salinity sea ice would expand with the increase of temperature and cause the change of scatterer structure to reduce the coherence. From [38], it can be inferred that the density of low-saline ice decreases with temperature in a nearly linear relationship with slope 1.6 × 10^−4^ kg/m^3^/K, so the effect on fast ice deformation could be ignored. In addition, the temperature on 14 February 2018 was about 0 °C for the most of the day. The Bothnia Bay is a low salinity sea area and the salt water will not freeze when the temperature above −0.2 °C [9], so the melting of sea ice surface also caused the decrease of coherence value. Secondly, a snowfall had occurred during the interval of data acquisitions, and the coverage of snow could reduce the coherence of some areas. Finally, the winds have greatly affected the coherence value, as shown in Figure 7, the winds were dominated by south and southeast wind and the maximum gust reached 16.5 m/s. The movements of ice floes driven by winds have resulted in the serious decorrelation, and the impact and compression on the fast ice caused by the drift of ice floes have resulted in the deformation of fast ice to reduce the coherence.

### 5.2. Characteristics of Interferograms

**Figure 9:**
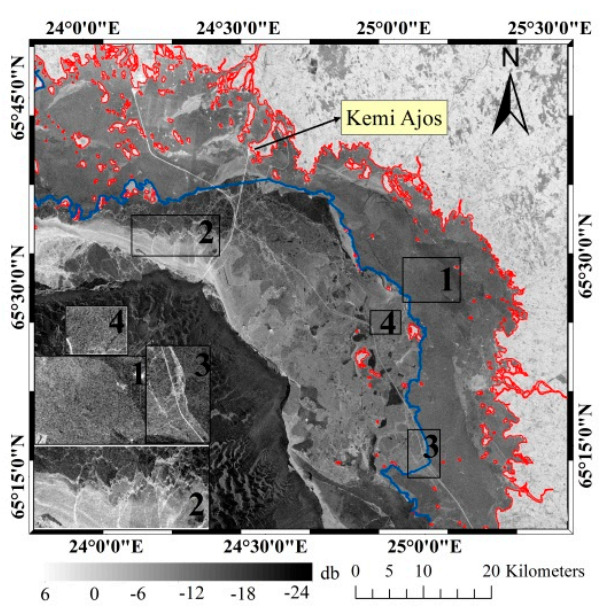
The SAR amplitude image corresponded to image pair A. The feature sites were corresponded to that in Figure 5a.

Some special interferometric fringes were enlarged and shown in Figure 5a and the amplitude image features corresponding to that were shown in Figure 9. Among them, the fringes were sparse and evenly distributed in site 1, and it reflected the similarity of the deformations [3]. The fringes in site 2 characterized with continuous disturbances and corresponded to the higher brightness values in the SAR amplitude image, which represented the locations of the ice ridges. In general, the discontinuous fringes implied the ice cracks or artificial ice roads. Referring to the SAR amplitude images, site 3 was a bright line in the SAR amplitude image and it represented an ice road. However, the SAR amplitude image corresponding to site 4 had no obvious features, but it was a gray line feature in the coherence map, so it represented an ice crack. For small-scale and refrozen ice cracks, it is difficult to identify them in SAR amplitude images by viewing geometry. However, the position of ice crack can be found by using the discontinuous interferometric phase. From the point of deformation stresses, the changes of fringe density and direction represented the changes of external forces that caused sea ice deformation. Deformations occur at a certain time suddenly, while the displacements obtained by InSAR were only the results of accumulation or counteraction for a long time [3]. As the temperature decreased again, the broken ice surface has refrozen. In addition, the drift direction of ice floes has been changed because of the change of wind direction. Both of them would have a certain impact on the interferograms.

### 5.3. Deformation Factors

As shown in Figure 6, the extreme displacement values occurred in the ice floes or consolidated ice (left of fast ice boundary), while the fast ice (right of fast ice boundary) maintained a relatively stable state in the deformation results of both descending and ascending orbits. As shown in Table 3, the displacement values obtained by the descending and ascending orbits data were 38 cm and 37 cm, respectively. It can be seen from Figure 6a,b that the deformation direction was mainly from east to west, which was also consistent with that in Figure 6c, and the uplift was dominated in the vertical deformation, as shown in Figure 6d.

It was the movements of ice floes driven by strong winds that were the main reason for the deformation of fast ice in study area. The continuous southeast winds existed on 9 and 10 February 2018 with speed over 12 m/s, as shown in Figure 7, which had resulted in the great deformations in ice floes or consolidated ice, and the maximum deformation in horizontal direction was about −1.08 m and the maximum vertical deformation was about −0.50 m. In terms of fast ice, however, the deformation dramatically decreased and the maximum horizontal and vertical deformations were −0.44 m and 0.16 m respectively.

Another factor for deformation was sea level tilt. Although the absolute sea level cannot affect the interferometric fringes, the sea level tilt might have a certain impact on the interferometric phase [8]. Fringes can change by sea level differences according to ∆R=φ/2k, where φ is the interferometric phase, and k=2π/λ, where *λ* is the radar pulse wavelength [7]. In this paper, sea level data from Kemi Ajos (24.51° E, 65.67° N) and Oulu Toppila (25.42° E, 65.04° N) were collected, as shown in Table 4. The maximum relative change in sea level was 2.9 cm with a distance between the stations around 82 km. Hence, the relative changes of sea level had little influence on the deformations in this study.

The low salinity ice will expand with increasing temperature at temperatures beneath about −5 °C, and the expansion coefficient is 10^−4^/°C [38]. In this paper, however, the deformation caused by thermal expansion can be ignored. Therefore, two conclusions can be drawn from the analysis. (i) The main reason for the fast ice deformation was the shear stress caused by the drift of ice floes under the strong southwest wind and the deformation mainly occurred in the horizontal direction from east to west. (ii) There was existing consolidated ice in the periphery of the fast ice, which played a protective role on the fast ice and weakened the influence of the drift or compression of ice floes.

### 5.4. Comparison with Previous Works

The researches that utilize InSAR technique to investigate the fast ice in Baltic Sea are few. Dammert, P.B.G. et al. [9] have detected the deformation of fast ice in Baltic Sea by using three scenes (24, 27 and 30 March 1992) ERS-1 SAR data. The results showed that the maximum displacement in LOS direction occurred on the ice sheet cut off by the tracks of two icebreakers, and the deformation value was 94 cm. However, the study area in this paper was not affected by the icebreaker, and there were no large ice surface cracks, so the deformation results were smaller.

Berg, A. et al. [7] have utilized four scenes Cosmo-SkyMed SAR data, two interferometric pairs (15 and 16 March 2012; 31 March and 1 April 2012), to study the sea ice in the north of the Bothnia Bay. The results showed that some ice floes moved northward at a speed of 100 m/day, impacting and squeezing the landfast ice, in one day time. And the deformation of landfast ice in LOS direction was about 4.7 cm over a distance of 1800 ± 25 m. The deformation obtained in this paper was about 5.3 cm corresponded to the same position in [7]. The same study area was the northern part of Bothnia Bay, in which many islands or reefs locate and the sea water is shallow near the shore. Attaching to those islands and reefs, fast ices were more stable and had small deformations, so the displacement value in 12 days was not very different from that in one day.

Marbouti, M. et al. [8] have first used the Sentinel-1A repeat-pass data to analyze the deformation trend and factors of fast ice in the Bothnia Bay. The result determined by two scenes (6 and 18 February 2015) descending orbits data has indicated that the displacement in LOS direction was 40 cm in the area of fast ice with 400 km^2^.This paper was similar to it in study area, experimental data and survey time. The results in this paper showed that the displacement values in LOS obtained by ascending and descending orbits data were 37 cm and 38 cm within the fast ice region of 960 km^2^ respectively.

Due to the lack of field measurements, we compared the results with previous works to verify the effectiveness and advantages of the results in this paper. As shown in Table 5, the different results of the same geographical location of Baltic Sea were compared in this paper. The results of this paper were consistent with those obtained by two previous researches, and the deformation difference in LOS direction was 0.6 cm and 3 cm respectively. It powerfully illustrated the accuracy and validity of the deformation results in this paper. Different from the previous studies, however, some new founds were obtained in this study. Because of the combination of the Sentinel-1A ascending and descending orbits data, the displacement value of fast ice in LOS direction was not only obtained, but also the vertical and horizontal deformations were achieved by the deformation transformed model, which are not available in previous studies. On this basis, the deformation trend and factors of fast ice has been further analyzed.

## 6. Conclusions

In this paper, the deformation of fast ice in Bothnia Bay of Baltic Sea has been studied using InSAR technology with the ascending and descending orbits data of Sentinel-1A. A method for automatically obtaining fast ice edge lines by combining interferometric coherence image and SAR amplitude image was proposed. According to the geometric relationship between multi-orbits deformation measurements, a deformation transformed model was established. It was used to successfully obtain the west-east and vertical deformations of fast ice through converting the displacement values in LOS direction.

The results showed that the maximum horizontal deformation was −44 cm and the maximum vertical deformation was 16 cm within the fast ice region of 960 km^2^ in the study area, during the study time from 2 to 16 February 2018. The main reason for the fast ice deformation was the shear stress caused by the drift of ice floes under the strong southwest wind and the major deformation was horizontal direction from east to west. In addition, the inner fast ice kept stable and its deformation was smaller due to the protection of consolidated ice.

One of the keys of InSAR technology is to obtain radar data with high coherence. In this study, the time baseline of interferograms was 12 days, which can not only effectively avoid the influence of some ice floes, but also effectively detect the developing trend and causes of fast ice deformation. Because of the lack of filed measurements, such as leveling, GPS measurements etc., however, the deformation results were not verified effectively. And the displacement values were accumulated or counteracted for a long time, which cannot accurately reflect the sudden or short-term sea ice changes, such as ice crack and ice drift. Recently, it has been proved that TerraSAR-X/TanDEM-X has considerable application prospects in the study field of sea ice [39,40]. In addition, an innovative radar detecting system has been proposed in the SWOT (Surface Water and Ocean Topography) mission of American [41,42], which is operating in a bistatic interferometric SAR system with small incidence angles and near-nadir swaths on both sides of the satellite track. Some Chinese scholars have suggested to introduce synthetic aperture technique and interferometry technology into radar altimeter and developed a new radar altimeter, i.e., three-dimensional imaging radar altimeter (CIALT, China Imaging Altimeter) [43,44]. All of these will provide an accurate and efficient means for obtaining the information of surface elevation and deformation quickly and in large area. Three-dimensional imaging radar altimeter will play an important role in the detection and application of sea ice in the future, so the following research work will focus on the detecting mechanism of sea ice, data processing and the inversing of sea ice parameters by using three-dimensional imaging radar altimeter.

## Figures and Tables

**Table 1:** Characteristic of Sentinel-1A interferometric mode [22].

Characteristic	Value
Swath width	250 km
Incidence Angle Range	29.1°–46.0°
Sub-Swath	IW1, IW2, IW3
Azimuth Steering angle	±0.6°
Maximum Noise Equivalent Sigma Zero (NESZ)	−22 dB
Radiometric Stability	0.5 dB (3σ)
Radiometric Accuracy	1 dB (3σ)
Phase Error	5°
Spatial resolution	5 × 20 m

**Table 2:** Summary of InSAR data.

	Image Pair A	Image Pair B
Acquisition dates	2 February 2018	4 February 2018
	14 February 2018	16 February 2018
Local time (UTC)	04:48	15:49
Orbit	Descending	Ascending
Look direction	Right	Right
Incidence angle	41.06°	36.12°
Normal baseline	13.05 m	61.65 m
Polarization	VV	VV
Relative orbit	51	87

**Table 3:** Deformation Results.

Directions	Entire Region ^1^		Fast ice Region ^1^	
	Deformation Range/m	Displacement Value/m	Deformation Range/m	Displacement Value/m
LOS (Descending)	−0.61–0.30	0.91	−0.18–0.20	0.38
LOS (Ascending)	−0.73–0.85	1.58	−0.07–0.30	0.37
West-East	−1.08–0.60	1.68	−0.44–0.26	0.70
Up-Down	−0.50–0.31	0.81	−0.03–0.16	0.19

^1^ The entire region refers to the overall sea ice, and the fast ice region refers to the right part of sea ice split by fast ice edge lines (as shown in Figure 6) obtained in this paper.

**Table 4:** Sea Level Changes.

Station	Image Pair A		Image Pair B	
	First Time	Second Time	First Time	Second Time
Kemi Ajos	−30.5 cm	4.4 cm	−17.6 cm	−5.1 cm
Oulu Toppila	−28.6 cm	3.4 cm	−16.1 cm	−3.0 cm

**Table 5:** Deformation Results Comparisons.

Deformation Results	Deformation in LOS	Max Vertical Deformation	Max Horizontal Deformation
Study by Berg, A. et al.	4.7 cm	—	—
Result in this paper	5.3 cm	1.3 cm	−25 cm
Deformation difference	0.6 cm	—	—
Study by Marbouti, M. et al.	40 cm	—	—
Result in this paper	37 cm	16 cm	−44 cm
Deformation difference	3 cm	—	—
